# P05-09 Associations of the physical activity-related injuries with selected variables in adolescents - results of the pilot study

**DOI:** 10.1093/eurpub/ckac095.076

**Published:** 2022-08-29

**Authors:** Peter Bakalár, Jaroslava Kopčáková, Lenka Tlučáková, Beáta Ružbarská, Monika Vašková, Viktoryia Karchynskaya

**Affiliations:** 1 Faculty of Sports, University of Presov, Presov, Slovakia; 2 Faculty of Medicine, Pavol Jozef Šafárik University in Košice, Košice, Slovakia; 3 University Medical Center Groningen, University of Groningen, Groningen, The Netherlands

**Keywords:** physical activity-related injuries, sports clubs, leisure, schools

## Abstract

**Background:**

Physical activity (PA) as health promotion tool is not one without adverse effects and adolescents with nonfatal physical activity-related injuries (PARI) may experience serious health consequences for the rest of their lives.

**Methods:**

As a part of the pilot study of the Health Behaviour in School-aged Children Study conducted in October and November 2021 in Slovakia, we surveyed 119 adolescents (53 girls; average age 12,6±2,0) for moderate-to-vigorous physical activity (MVPA), medically attended injuries (MAI), physical activity-related injuries in sports clubs (PARISC), physical activity-related injuries in leisure-time (PARILT) and physical activity-related injuries in schools (PARIS) and we measured their cardiorespiratory fitness (using 20-metre shuttle run) and their body composition (using InBody 230).

**Results:**

Out of 119 adolescents, 50 (42%) were attending sports clubs of which 27 (54%) had one or more PARI in sports clubs' activities in previous year, 50 adolescents (42%) had PARI in leisure activities and 15 (12,6%) in school activities. PARISC led to an average of 10 missed days from school or leisure-time activities. PARILT led to 7,2 missed days and PARIS led to 6,2 missed days. Spearman's correlations (n = 50 for PARISC and n = 119 for PARILT and PARIS) revealed associations between MAI and PARISC, PARILT and PARIS, but not between MVPA or 20-metre shuttle run laps and PARISC, PARILT and PARIS. Not surprisingly, percentage of body fat was negatively associated with the number of 20-metre shuttle run laps. In addition, results of crude linear regression models showed that frequency of MVPA was not associated with frequencies of PARISC (B coefficients (B)/95% CI: 0,03/-0,11-0,18), PARILT (B/95% CI: 0,04/-0,05-0,13) or PARIS (B/95% CI: -0,02/-0,07-0,02) among Slovak adolescents in our pilot study.

**Conclusions:**

Estimating the burden of PARI is important in advocating the need of directing sufficient resources to PARI prevention along with the PA promotion. Improvement and understanding of factors associated with PARI might be helpful in PARI prevention. In addition, it might, among other factors, play a role in promotion of active lifestyle in adolescence.

